# Pharmacological Treatment for Acute Traumatic Musculoskeletal Pain in Athletes

**DOI:** 10.3390/medicina57111208

**Published:** 2021-11-05

**Authors:** Alessandro de Sire, Nicola Marotta, Lorenzo Lippi, Dalila Scaturro, Giacomo Farì, Alfonso Liccardi, Lucrezia Moggio, Giulia Letizia Mauro, Antonio Ammendolia, Marco Invernizzi

**Affiliations:** 1 Physical and Rehabilitative Medicine, Department of Medical and Surgical Sciences, University of Catanzaro “Magna Graecia”, 88100 Catanzaro, Italy; nicola.marotta@unicz.it (N.M.); lucrezia.moggio@gmail.com (L.M.); ammendolia@unicz.it (A.A.); 2 Physical and Rehabilitative Medicine, Department of Health Sciences, University of Eastern Piedmont, 28100 Novara, Italy; lorenzolippi.mt@gmail.com (L.L.); marco.invernizzi@med.uniupo.it (M.I.); 3 Physical and Rehabilitative Medicine, Department of Surgical, Oncological and Stomatological Disciplines, University of Palermo, 90100 Palermo, Italy; dalila.scaturro@unipa.it (D.S.); giulia.letiziamauro@unipa.it (G.L.M.); 4 Motor and Sports Sciences, Department of Sciences and Biological and Environmental Technologies, Salento University, 73100 Lecce, Italy; dr.giacomofari@gmail.com; 5 Department of Biomedical Sciences for Health, University of Milan, 20122 Milan, Italy; alfonsoliccardi91@gmail.com; 6 Translational Medicine, Dipartimento Attività Integrate Ricerca e Innovazione (DAIRI), Azienda Ospedaliera SS. Antonio e Biagio e Cesare Arrigo, 15121 Alessandria, Italy

**Keywords:** pain management, athletic injuries, trauma, return to sport, sport medicine, rehabilitation

## Abstract

Pain management is a crucial issue for athletes who train and compete at the highest performance levels. There are still evidence gaps for the use of analgesics for sports injuries despite the growing interest in training and competition settings. However, high-quality research is needed to determine the most appropriate and optimal timing and formulations in non-steroidal anti-inflammatory drug and opioid management, particularly given the strictness of anti-doping regulations. Indeed, the role of pharmacological therapy in reducing acute traumatic pain in athletes should still be addressed to minimize the timing of return to sport. Therefore, the aim of this comprehensive review was to summarize the current evidence about pain management in the setting of acute injury in elite athletes, providing the most informed strategy for pain relief and performance recovery.

## 1. Introduction

Growing attention has been paid to the promotion of physical activity and a healthy lifestyle over the past decades, with an improved number of initiatives promoting sports in both healthy subjects and patients suffering from various diseases [1,2,3,4]. As a result, a constant increase has been registered in the prevalence of both elite and recreational athletes, particularly in youth [5,6]. Apart from the widely noted positive effects of physical activity on cardiovascular and musculoskeletal health [1], the increase in the number of athletes has increased the incidence of sport-related musculoskeletal injuries, with relevant issues on sanitary costs and time lost from sport [7,8,9].

The International Olympic Committee (IOC) has recently defined sports injuries as new or recurring musculoskeletal complaints occurred during competition or training and requiring medical attention [10]. However, acute sport-related musculoskeletal injuries are characterized by a large heterogeneity in epidemiology and clinical presentation based on the sport performed [11,12]. Additionally, it has been reported that acute traumatic musculoskeletal injuries represent the 10–19% of all acute injuries treated in the emergency department [13,14]. Sports-related injuries still represent a critical issue in sports medicine, despite the large attention paid to prevention programs [15,16]. To date, the negative effects of pain on training and physical function lead to a psychological and economic burden for athletes and their teams [17,18].

In this situation, an individualized and patient-tailored approach, including pain management, physical therapy, and rehabilitation, has been recommended to manage sports-related musculoskeletal injuries [10,19,20,21,22,23]. In 2016, Ahmadi et al. [24] assessed the pharmacological approach to traumatic injuries, highlighting the need for an age-specific individualized treatment. However, it should be noted that sports athletes usually require a tailored management to enhance the rehabilitation and the return to play (RTP) [24]. Accordingly, Zideman et al. [23] confirmed that a multitarget approach is crucial for elite athletes in terms of pain management. However, the authors included both nonpharmacological and pharmacological approaches, without focusing on acute traumatic injuries [23].

To the best of our knowledge, the role of pharmacological therapy in reducing acute traumatic pain in sportsmen should be addressed to optimize pain management, minimizing the timing of RTP.

Thus, by the present comprehensive narrative review, we aimed to summarize the state of the art about the pharmacological treatment for acute traumatic musculoskeletal pain in athletes, in order to enhance knowledge on this subject, and to guide physicians in the common clinical practice, management, and rehabilitation of these subjects.

## 2. Main Acute Traumatic Musculoskeletal Pain in Athletes

Musculoskeletal injuries represent the most common sports-related injuries [25], though the characteristics of specific sports and the physical stresses related to sports activities significantly affect the prevalence of the different types of injuries [14]. Therefore, an adequate assessment of sport-specific demands is particularly important to guide physicians not only in specific diagnoses but also in the prescription of a multitarget and multimodal therapeutic plan including pain management, sport-specific rehabilitation, and prevention of re-injuries [26].

Conversely, several athletes’ characteristics should be taken into consideration to provide a precise diagnosis, including the main risk factors affecting injury risks such as age, gender, and level of play [27,28,29]. In particular, it has been reported that ankle sprains and knee injuries are more common in women, probably due to higher estrogen levels, higher body fat mass, lower muscle mass, greater flexibility, and a wider pelvis [27,28,30]. At the same time, chronic and overuse injuries are most common in older athletes, given the loss of elasticity and the less effective reparation mechanisms in tendons and muscles [31]. Accordingly, the level of play might significantly affect injury risk due to the number of hours of play per week, and the intensity of training and competitions [32]. Therefore, clinicians should be aware of the potential implications of a patient’s characteristics, guiding a precise diagnostic process aimed at supporting a precise diagnosis that represents the starting point for an individualized and tailored pain management.

**Figure 1:**
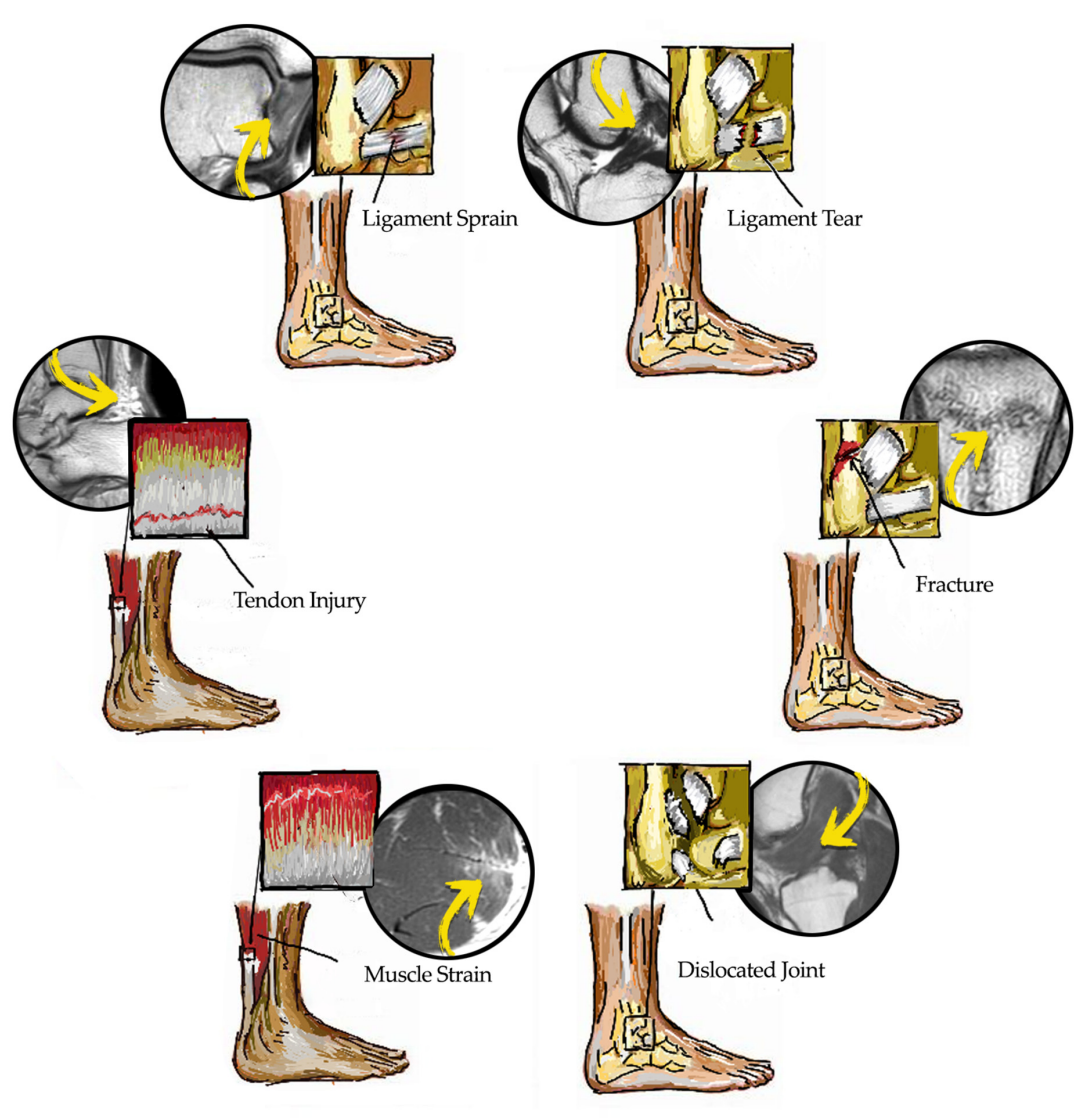
Main sports-related traumatic musculoskeletal injuries (graphical model and magnetic resonance imaging).

In these scenarios, acute traumatic musculoskeletal injuries are characterized by a sudden trauma to the tissue, commonly related to a specific identifiable event during the sports activity. Acute sport-related injuries might be classified according to the tissues involved in the injury (e.g., bone, ligament, muscle, tendon, joint,) and the type of injury (e.g., fracture, dislocation, sprain, or strain) [33] (See Figure 1 for further details on ankle injuries as an example).

In further detail, the main painful sports-related traumatic injuries could include:

- *Ankle sprains:* These represent very common sports-related musculoskeletal injuries, especially in team sports [34]. An incidence of 3.1 per 1000 ankle sprains per season in elite athletes [35,36] is estimated. Recovery time and type of rehabilitation protocol are structured based on the severity of the injury. Unfortunately, the injury significantly predisposes the athlete to recurrent ankle sprains [37].
- *Knee injuries*: Anterior cruciate ligament (ACL) injuries are among the most disabling sports-related issues and occur in a range of 29 to 38 per 100,000 athletes [38,39]. However, other structures might be torn or overstretched during a knee sprain, resulting in injuries of posterior cruciate ligament, medial collateral ligament, lateral collateral ligament, or capsular sprain [40]. Nonetheless, meniscus injuries represent 8% of all seasonal injuries in professional football, since it is a sport characterized by pivoting and cutting movements [41].
- *Muscle injuries*: Muscle injuries represent one-third of sport-related injuries in soccer players and 92% of them affect hamstrings, adductors, quadriceps, and calf muscles [42]. The pathological process can be characterized by direct trauma (muscle contusion) caused by a direct impact on soft tissues [43], or by indirect trauma (contraction-induced injury) due to severe mechanical stress on muscle fibers. In particular, the principal cause of indirect injuries is an excessive eccentric contraction or overstretching of the muscle, frequently related to rapid acceleration or deceleration [33,44]. To date, several classifications have been proposed to better characterize these injuries, and treatment and prognosis are based on the following classification [45,46,47].
- *Tendon injuries*: the most common acute tendon injury is the tendon rupture, due to an acute traumatic single event that leads to a singular macro-trauma on previously healthy tissue, not involved in chronic inflammation or a degenerative process [48]. The most common tendon involved is the Achilles tendon [49], and male and older athletes seem to be the most affected, reaching an incidence of approximately 40/100,000 persons per year [50]. Despite several approaches being proposed, surgical intervention is the most used in clinical practice, followed by an early functional rehabilitation program with evidence of a significant reduction in risk of recurrence [51].
- *Upper limb injuries*: Shoulder dislocations represent relatively common sports-related injuries, consisting 3.6% of all injuries in high school athletes, with an overall rate of 2.04 per 100,000 athletes [52]. They may occur when two or more bones are forced out of their normal position resulting in an abnormal and permanent separation in the joint. The shoulder dislocation represents 54.9% of dislocation in athletes [52,53]. Due to their traumatic etiology, dislocations are more frequent in sports with high risk of falling, such as rugby, hockey, and wrestling [54]. On the other hand, in the elite athlete, rotator cuff injuries can occur with acute episodes of direct contact trauma, a fall on an outstretched arm, and represent almost half (47%) of overall shoulder injuries per single season [55]. Medial ulnar collateral ligament (MUCL) injuries are described primarily as chronic progressive injuries but are underestimated as acute lesions in young athletes who play overhead sports [56].
- *Fractures*: Sport-related fractures, 5–10% of injuries in athletes, are very painful conditions, commonly resulting from a sudden trauma especially in contact sports or in sports with high risk of falls [57,58]. In this context, pain management plays a key role and requires specific strategies depending on the site of the fracture [59].

Therefore, alongside an adequate pain management, an optimization of the functional recovery and a safe RTP should be taken into consideration for athletes with sports-related injuries.

## 3. Analgesic Pharmacological Approach

The analgesic pharmacological approach historically represents the cornerstone of pain management for acute traumatic musculoskeletal injuries [25,60]. However, it should be noted that the pharmacological approach should be considered just one component in an integrated and multidisciplinary approach aimed at reducing physical impairment and optimizing functional recovery and RTP [61,62,63,64]. The IOC Consensus Statement [10] recommended that analgesic drug prescription should be performed targeting the lowest effective dose for the shortest period. Therefore, particular attention should be paid to minimize adverse risks and to achieve pain relief; furthermore, in the case of ineffective or intolerance to treatment, the drug should be discontinued [10].

Tissue location, type of injury, and pain severity significantly influence the treatment approach [65]. In this context, a recent review [23] underlined those minor injuries that might often be treated by non-opioid analgesic medication and non-pharmacological treatment; however, the effectiveness of this approach in different pathologies has not been deeply studied. In minor injuries, when same-day RTP is considered, oral or local analgesic drugs are routinely used according to the scientific literature that showed a positive effect [25,66,67,68,69,70,71,72,73,74,75].

Furthermore, when prescribing analgesic drugs, it is necessary to carefully consider different mechanisms of action and safety, as different pharmacological agents may be associated with different side effects [76]. According to the World Health Organization (WHO) pain ladder [77], non-narcotic analgesics may be used to manage mild to moderate pain. Nevertheless, the combination with narcotic analgesics may be performed to manage severe pain to obtain synergic effects [76]. To date, several options are currently available in the pharmacological non-invasive approach in acute traumatic injury, including paracetamol [78,79,80,81], non-steroidal anti-inflammatory drugs (NSAIDs) [80,82,83,84,85], and opioids [79,80,81,86,87,88].

- *Paracetamol*: one of the most common drugs routinely used for mild and moderate pain alone or combined with other pharmacological and non-pharmacological interventions [89]. In accordance with the WHO Pain Ladder Recommendations [89], it should be considered as a first-line treatment in mild pain. Furthermore, the safety of paracetamol at up to 3 g per day is well documented with studies reporting adverse effects compared to placebo [90,91]. In addition, studies comparing paracetamol to NSAID for treating pain after acute sport-related injuries did not report significant differences in term of effectiveness [92]. Similarly, a combination of paracetamol and NSAIDs might be more effective in pain relief, even if side effects might be more frequent [93].
- *NSAIDs*: This pharmacologic treatment is highly supported by the literature for pain management [80,82,83,84,85,94,95,96]; however, specific interventions including the exact time, dose, and duration of specific NSAIDs targeting a specific acute traumatic injury are still lacking. Apart from these limitations, NSAIDs are currently the most prescribed drugs in sport-related injuries, but another crucial issue is represented by the administration modalities.

In this context, a recent Cochrane review showed that topical diclofenac and ketoprofen could be effective in pain relief of acute sprains and strains [97]. Moreover, the intradermal absorption significantly minimizes the risk for adverse events, and the approximate benefit of 50% of pain relief after 1 week makes this treatment suitable as a first-line treatment for minor acute injuries [97,98]. To date, ketorolac is the most supported NSAID in acute pain management, as confirmed by a recent meta-analysis [99] highlighting the role of ketorolac in severe pain relief and an adequate safety profile if dismissed in 5 days. On the other hand, no randomized controlled trials (RCTs) have been performed supporting the ketorolac effects when compared to different NSAIDs. A recent report underlined a potential increase in the risk of post-traumatic hemorrhage and acute traumatic injuries, underlining that NSAIDs should be carefully administered in these subjects [100]. NSAIDs should be avoided in the first 72 h from brain concussion given the increased risk of bleeding [101]. In accordance, since inflammation has a crucial role in the first processes at the basis of tissue healing in the skeletal muscle system [68,102,103,104,105], the anti-inflammatory pharmacological approach has been recently questioned [68]. Moreover, NSAIDs mediate their pharmacological effects by the inhibition of prostaglandin synthesis in the COX pathway; therefore, a careful prescription of these drugs should consider the evidence highlighting the potential negative effects in bone, muscle, ligament, and tendon healing [68,102,103,104,105]. However, no studies assessed the long-term effects of NSAIDs in athlete global health, tissue overload on the kinetic chain continuum, injury recurrence, or complications related to pain relief effects [23].

- *Opioids:* These drugs should be prescribed in sports for major traumas that could be related to a high risk of bleeding and severe pain. Indeed, in these cases, NSAIDs did not represent a suitable option according to the WHO pain ladder recommendations [89]. In this context, the NICE guideline for major trauma in adults [106] supported the use of intravenous morphine as the first-line analgesic in the hospital or pre-hospital setting to achieve adequate pain relief without affecting blood coagulation. The IOC recommended that opioids should be considered for athletes only in case of severe pain with the initial prescription not exceeding 5 days [107]. However, longer prescriptions have been reported despite the critical issue of risks of opioid dependence or addiction.

Rehabilitation might play a crucial role as a synergistic approach with the pharmacological therapy to reduce acute traumatic musculoskeletal pain. In this situation, instrumental physical therapies might be considered a complementary approach in both acute and chronic pain [108,109]. In addition, optimal pain management is a cornerstone of a proper rehabilitation plan, thus avoiding the psychological burden related to musculoskeletal pain and minimizing the fear of movement that might significantly affect the time of RTP and reinjury risk [110,111,112].

Moreover, treatment reducing deconditioning and enhancing physical performance should be considered even in the early stages of acute traumas [113]. Thus, the term “rehabilitation pharmacotherapy” has been recently introduced to characterize a specific pharmacological approach aimed at achieving the highest physical performance, physical function, and quality of life; a rehabilitative intervention that aims to influence the therapeutic approach, calming the multi-drug management [114]. Despite the studies supporting this approach mainly focused on older people [114,115], “rehabilitation pharmacotherapy” should be seriously considered in the pain management of sports athletes. Therefore, the pharmacological approach should not interfere with rehabilitation and RTP [114,115]. However, more evidence is needed to fully support this approach in athletes.

## 4. Invasive Pain Management: What Role for Infiltrations in Athletes?

Mini-invasive procedures are commonly used in clinical practice for chronic pain [109,116,117,118], but they are still not adequately investigated and performed for acute traumatic sports-related injuries [72,119,120]. In this situation, local anesthetic injections are frequently performed to ensure an anticipated or immediate RTP in elite athletes [121]. Drakos et al. [121] reported the effects of ultrasound-guided local anesthetic injections within 1 h of competition in patients suffering from muscle injuries and ankle sprains, demonstrating a high safety profile, and supporting their role in anticipated RTP.

To date, there is a lack of evidence on the impact of local anesthetic injections in acute sports-related injuries [122]. It is crucial that the type of the treatment should depend on the site of the injury [123,124,125]. However, the majority of evidence supporting corticosteroid injections for musculoskeletal pain involves non-athletes [126,127,128,129,130]. In addition, the corticosteroid effects in pain relief in acute sport-related injury are not fully supported by the current literature due to the potential interaction in physiological molecular pathways underpinning tissue healing [122,131,132].

Hyaluronic acid (HA) injection, notably, represents an effective and safe option in the treatment of articular pain in athletes, with recent evidence suggesting a potential effect in accelerating RTP [122,133]. To date, the Osteoarthritis Research Society International classification [134] reported that HA injections might be a recommended mini-invasive procedure to treat even early osteoarthritis in older athletes [122].

On the other hand, in young athletes, HA injection might have a role in multifactorial pain syndromes involving articular damage or cartilage involvement. In particular, the recent review by da Costa [135] supported HA injection in a multimodal treatment of patellar chondropathy. Moreover, given the lack of doping issues [136], HA injection represent a feasible and safe therapy for sport-related injuries in elite athletes [122,133]. However, in severely injured athletes, who have no benefit from conventional therapies, mini-invasive procedures should be taken into consideration [131,132].

Another technique that might be performed for acute pain in athletes is regional anesthesia, a procedure strictly linked to the type of injury; in particular, forearm nerve blocks or axillary blocks might be effective in pain management of hand and forearm fractures [137,138]. In addition, distal radius fracture management during reduction might involve hematoma block (achieving good results in pain relief) [58]. Similarly, sciatic nerve blocks, adductor canal nerve blocks, fascia iliaca nerve blocks, and femoral nerve blocks have been demonstrated to be effective in specific pathologies to achieve adequate analgesia and reduce opioid medications after acute traumatic injuries requiring surgery [58,139,140,141,142]. Despite the promising effects of nerve block injections, some authors reported that regional anesthesia might mask compartment syndrome onset, therefore these procedures should be carefully performed [58].

However, it should be noted that, though nerve block might improve pain management and reduce oral medication [143,144,145], multimodal analgesia has been strongly recommended [146]. Therefore, mini-invasive procedures should be considered in a combination of pharmacological strategies to potentially reduce the side effects of monotherapy and improve pain management in acute injuries [146].

Moreover, infiltrations should always be framed within a comprehensive, multidisciplinary approach, including not only other pharmacological approaches but also nonpharmacological ones. Indeed, physical therapy might play a key role in pain control with growing evidence supporting its effects if combined with mini-invasive rehabilitative approaches [109,112,117,147]. To date, several reports suggested that physical activity might modulate pain perception modulating central nervous system excitability, and improving psychological constructs associated with pain [148,149].

On the other hand, rehabilitation might have several synergic effects with mini-invasive procedures, especially in athletes [150]. In more detail, pain control during rehabilitation might improve muscle tension, encourage realignment of the body, and prevent the fear of movement that affects functional outcomes and reinjury risk, potentially enhancing rapid recovery and RTP [151,152,153]. At the same time, physical therapy might induce pain relief by decreasing inflammation, increasing mobility, and decreasing overall pain levels with a high safety profile [112,154,155]. Moreover, considering the crucial issue of RTP in athletes, any therapeutic pathway should be integrated with treatment that enhances physical conditioning, including physical therapy, and retraining exercise [156,157].

In conclusion, rehabilitation represents a suitable therapeutic option in multimodal pain management (including infiltrations) for the management of musculoskeletal pain in athletes. However, these mini-invasive approaches should be safe, appropriate, and patient-tailored to optimize functional recovery and RTP in a comprehensive multimodal rehabilitation plan.

## 5. Return-to-Play after Trauma

RTP is crucial for elite athletes after an acute trauma, given the economic and competitive entanglements associated with absence for professional players [158]. Injury management should include proper pain management as a key step of a tailored intervention to enhance the RTP program [159]. Therefore, an evidence-based epidemiological report might guide physicians in the prognostic assessment to approach concerns from players, coaches, managers, media, and agents regarding RTP [160].

RTP may change according to the anatomical site and severity of the acute traumatic pain:

- *Ankle sprains:* Physicians should evaluate specific movements to determine appropriate RTP following an ankle sprain. Athletes should use a drop test to estimate the sportive gesture evaluation, considering muscle strength, proprioceptive balance, and joint range of motion [161]. In summary, appropriate RTP must be based on objective and scalable assessments that inform professionals about the future risk of injury. The experts’ consensus indicates that RTP must focus on the sport gesture that the athlete requires in practice [161]. Unfortunately, due to the lack of evidence, each practitioner needs to establish an objective threshold for assessing a tailored RTP [162].
- *Knee injuries:* About half of athletes return to competitive sport after primary ACL reconstruction [163]. Regardless of treatment, the RTP rate is affected by factors including specific sport demands and regional differences. RTP rates are lower after revision ACL reconstruction than after primary surgery [164]. Interestingly, the duration of RTP after an ACL reconstruction has a high variability in male professional football players, but, independent of this time, only a minority of athletes return to their pre-injury level 1 year after surgery [165]. However, the mean lay-off time in professional football for all Medial Collateral Ligament (MCL) injuries is 23 ± 23 days [166] given that MCL injuries can be managed conservatively, though grade III MCL injuries or involvement of the deep MCL and/or the posterior oblique ligament are associated with longer recovery time. Lastly, athletes requiring lateral meniscus treatment have longer recovery times and lower RTP rates than athletes who require medial meniscus treatment [167].
- *Muscle injuries:* Acute hamstring injury is the most frequent non-contact muscle injury in sports involving high-speed running, with a consistently high incidence and high reinjury risk [42,168,169]. Given the high heterogeneity of injury location and severity, time to RTP after acute hamstring injuries varies substantially from an average of 11.3 days to 50 weeks [170]. Among professional football players, the mean lay-off time was shown to be around 20 days [167]. On the other hand, total proximal hamstring ruptures might represent the highest grade of muscle injury, requiring surgery; therefore, RTP duration sensibly increases and is generally allowed after 6–9 months [171]. Intriguingly, there is currently no strong evidence that Magnetic Resonance Imaging (MRI) could be a predictive factor for RTP [172,173]. However, RTP is not a major issue in acute hamstring injuries because close to 100% of athletes RTP after an injury [174].
- *Tendon injuries:* The Achilles tendon is one of the most injured tendons in athletes involved in running and jumping activities [174]. It has been estimated that between 10 and 86% of athletes have RTP after 12 weeks of treatment for Achilles tendinopathy. However, up to half of athletes have a recurrence of Achilles tendinopathy after RTP [174]. After Achilles tendon rupture, 29–87% of athletes return to their pre-injury level [175]. Nevertheless, rehabilitation exercises are the cornerstone of RTP in Achilles tendinopathy, given that those who follow a standardized load progression have fewer incidences of recurrence compared with those who do not follow a progressive loading program [176]. Apart from these findings, permanent deficits in calf muscle strength and tendon elongation can be very common after Achilles tendon rupture [177]. Therefore, it is particularly important that athletes with Achilles tendinopathy undergo a full progressive loading program prior to clearance to RTP [176]. On the other hand, there are no milestone-based criteria for RTP following Achilles tendon rupture. The time-based criteria for non-contact sports are resumption from 16 weeks following injury and for contact sports from 20 weeks after injury [178].
- *Upper limb injuries*: Shoulder dislocations represent the most common site of dislocation in athletes. Nevertheless, little evidence exists regarding the physical RTP criteria of the shoulder after injury [179]. In more detail, nonoperatively treated shoulder instabilities that sustained shoulder subluxations returned after an average of 3.6 weeks, compared with 7.6 weeks in those who sustained a shoulder dislocation [179]. Players experiencing shoulder dislocations were found to miss more time before RTP and were more likely to undergo surgical intervention compared with those who experienced a subluxation [180]. A recent review [181] demonstrated no overall difference in the rate of RTP or patient-reported outcomes following arthroscopic Bankart repair, the Latarjet procedure, and open stabilization [182]. Return to the same level of play after surgical repair of full-thickness tears in professional overhead athletes has been unpredictable and often this kind of injury is career-ending; arthroscopic repair of full-thickness tears in professional baseball players allowed 83% to return to play, but few with pre-injury levels of competition [55]. Regarding MCUL, it is well known that operative treatment represents the gold standard for professional players, but, to date, there is no consensus on which approach (i.e., conservative or surgical) represents the best choice for high-performance-demand athletes given the poorly predictable RTP [56].
- *Fractures*: Sports-related fractures may have critical implications for athletes. Fracture management tends toward preserving soft tissue, which is critical for an early recovery in the athletic population [58,183]. Given the breadth of circumstances faced during athletic activity, there is no patterned algorithm for defining a player’s readiness to RTP after a fracture. Nevertheless, an athlete should be pain-free, neurologically unimpaired, and without deficits of strength or range of motion before returning to sport [184,185].

## 6. Antidoping Issues in the Pain Management of Athletes

Pain management has constantly influenced athletic conditions, thus making necessary the publication of a prohibited substances list by the World Anti-Doping Agency (WADA) [186].

In this context, doping indicates the presence, use, possession, or trafficking of a prohibited substance. Evidence suggests that currently banned narcotic analgesics (opioids) and cannabinoids are not ergogenic but ergolytic [84,186]. Indeed, both can be prescribed by a physician for pain control, but both can be obtained illegally and carry potentially serious health risks, including addiction.

In contrast, the most used analgesics, including non-steroidal anti-inflammatory drugs, acetaminophen, local anesthetics, and tramadol are not forbidden according to the WADA list of prohibited substances [84,186]. However, potential ergogenic effects of NSAIDs in sports performance have been recently reported, and, unfortunately, a significant difference between the use of NSAIDs in-competition vs. out-of-competition has been demonstrated, probably related to the postulated effects in improving physical performance [186,187,188,189,190,191]. Interestingly, performance enhancement seems to be related to the widely noted antalgic effect, which might improve exercise-induced pain level tolerance with consequent positive effects in sports performance [190]. Despite these considerations, the ergogenic effects of NSAIDs have not been supported by strong evidence and these molecules are still not considered forbidden [186].

Moreover, one of the most used methods to control pain is the injection of a local anesthetic, which raises little controversy in sport compared to opiate management, a currently contentious topic perhaps given the epidemic of opiate abuse in some regions of the world [190]. Indeed, although tramadol is not on the list, it may sometimes be abused, such as in cycling [191]. There is a solid argument that all opioids and cannabinoids should be available to the practitioner, but with a remarkably close window for managing these substances in elite competitors [192,193].

## 7. Conclusions and Future Perspectives

In this comprehensive review, we summarized the state-of-the-art in pharmacological intervention efficacy on the pain management of acute sport-related injuries in athletes. Moreover, we highlighted the need for an effective assessment and management of musculoskeletal disorders to obtain a prompt RTP. Intriguingly, there is still a large gap of knowledge in drug-induced pain relief in athletes in terms of kinematic and physical performance.

However, the optimal strategy to manage sport-related injuries should include not only pharmacological interventions but also tailored exercise prescription and load management and rehabilitation. These interventions should be performed by medical, sports science, and technical staff to provide the most effective management of sport-related acute injuries.

To date, evidence about the impact of pharmacological approaches on acute traumatic injuries in terms of kinematic outcomes, RTP, and long-term well-being of athletes is far from being fully established. Therefore, further high-quality studies (both systematic reviews and RCTs) focusing on the most effective rehabilitative and pharmacological interventions to treat acute sport-related injuries are warranted. This could provide evidence to guide physicians in the comprehensive clinical and rehabilitative management of acute traumatic sports-related injuries.

## Data Availability

Not applicable.
